# Changes in the epidemiological characteristics of human brucellosis in Shaanxi Province from 2008 to 2020

**DOI:** 10.1038/s41598-021-96774-x

**Published:** 2021-08-30

**Authors:** Cui-hong An, Zhi-guo Liu, Shou-min Nie, Yang-xin Sun, Suo-ping Fan, Bo-yan Luo, Zhenjun Li, Ji-ru Xu

**Affiliations:** 1 Department of Microbiology and Immunology, School of Medicine, Xi’an Jiaotong University, Xi’an, 710061 Shaanxi Province China; 2 Department of Plague and Brucellosis, Shaanxi Center for Disease Control and Prevention, Xi’an, 710054 China; 3 State Key Laboratory of Infectious Disease Prevention and Control, National Institute for Communicable Diseases Control and Prevention, Chinese Center for Disease Control and Prevention, Beijing, 102206 China

**Keywords:** Infectious diseases, Bacterial infection

## Abstract

In the present study, surveys of case numbers, constituent ratios, conventional biotyping, and multilocus sequence typing (MLST) were applied to characterize the incidence rate and epidemiological characteristics of human brucellosis in Shaanxi Province, China. A total of 12,215 human brucellosis cases were reported during 2008–2020, for an annual average incidence rate of 2.48/100,000. The most significant change was that the county numbers of reported cases increased from 36 in 2008 to 84 in 2020, with a geographic expansion trend from northern Shaanxi to Guanzhong, and southern Shaanxi regions; the incidence rate declined in previous epidemic northern Shaanxi regions while increasing each year in Guanzhong and southern Shaanxi regions such as Hancheng and Xianyang. The increased incidence was closely related to the development of large-scale small ruminants (goats and sheep) farms in Guanzhong and some southern Shaanxi regions. Another significant feature was that student cases (n = 261) were ranked second among all occupations, accounting for 2.14% of the total number of cases, with the majority due to drinking unsterilized goat milk. Three *Brucella* species were detected (*B. melitensis* (bv. 1, 2, 3 and variant), *B. abortus* bv. 3/6, and *B. suis* bv. 1) and were mainly distributed in the northern Shaanxi and Guanzhong regions. Three known STs (ST8, ST2, and ST14) were identified based on MLST analysis. The characteristics that had not changed were that *B. melitensis* strains belonging to the ST8 population were the dominant species and were observed in all nine regions during the examined periods. Strengthened human and animal brucellosis surveillance and restriction of the transfer of infected sheep (goats) as well as students avoiding drinking raw milk are suggested as optimal control strategies.

## Introduction

Brucellosis is a widespread zoonotic disease caused by species of the genus *Brucella*, which are small, gram-negative, nonmotile, facultative intracellular coccobacilli^1^. Brucellosis has been successfully controlled in many developed countries through the implementation of multifaceted control strategies that include vaccination and test-and-slaughter of infected livestock^2^. However, at least 500,000 cases of human brucellosis are reported annually worldwide^3^. Direct contact with infected animals and the ingestion of contaminated dairy products are the most common transmission routes. The elimination of infected animals is the most effective prevention strategy for brucellosis^4^. Fever, sweating, and muscle joint pain are common manifestations of human brucellosis, and the disease can progress to a chronic condition with the appearance of severe complications^5^. Brucellosis represents a significant threat to animal husbandry and results in enormous economic losses. Furthermore, brucellosis is one of the major public health concerns in China, and the number of human brucellosis cases has increased dramatically^6^. Shaanxi Province is located in Northwestern China. There are eight provinces bordering Shaanxi—Henan, Hubei, Chongqing, Sichuan, Gansu, Ningxia, Inner Mongolia, and Shanxi Provinces—and the majority are epidemic regions for human brucellosis^7^. Shaanxi Province has 14 districts (cities and counties) and 107 counties (cities, districts) under its jurisdiction. The region is divided into northern Shaanxi, Guanzhong, and southern Shaanxi, of which northern Shaanxi is dominated by agriculture and animal husbandry.

Based on previous reports, Shaanxi was divided into general brucellosis epidemic areas in China^8^. From 2005 to 2018, a total of 12,671 confirmed human brucellosis cases were reported in Shaanxi Province, and the average annual incidence reached 11.50/100,000, which was higher than the national average incidence in 2018 (2.73/100,000)^9^. The latest study focuses on the spatial and temporal distributions and model for risk prediction of human brucellosis in Shaanxi Province between 2005 and 2018. This study showed that human brucellosis cases were mainly distributed in the Shaanbei upland plateau before 2008 and then slowly extended towards the southern region with significant seasonal fluctuations^10^. Multiple climatic factors (air temperature, sunshine duration, rainfall and relative humidity, etc.) have potent pertinence to the transmission of human brucellosis with seasonal fluctuations^10^. However, comprehensive analysis of the epidemic situation, incidence rate of regions, specific demographic and seasonality features of human brucellosis, and characterization of the species/biovars, geographic distribution and genetic profile of *Brucella* strains isolated in this province is necessary to formulate targeted control measures.

Multilocus sequence typing (MLST) is a robust tool used to investigate global epidemiological and phylogenetic relationships in bacterial populations. Combining data from sets of multiple gene fragments can be highly discriminatory while retaining signatures of long-term evolutionary relationships^11^. Moreover, a previous study found that MLST is suitable for discrimination at the species level, and the method has been used to perform population-level investigations of *Brucella* isolates^12^. Notably, a significant advantage of MLST is that sequence data can be shared between laboratories and individuals, thereby promoting the exchange of molecular typing data for the global epidemiology of pathogens^13^. Therefore, our study combined epidemiological indices, conventional biotyping, and the MLST approach to investigate the epidemiological characteristics of human brucellosis and the structural characteristics of *Brucella* populations to formulate an available strategy for control and prevention of human brucellosis in Shaanxi Province.

## Materials and methods

### Ethics statement

This research was carried out according to the principles of the Declaration of Helsinki. The study protocol was approved by the Medical Ethics Committee of the Shaanxi Province Center for Disease Control and Prevention. The ethics approval number is 2017-001-01. Informed consent was obtained from all patients prior to diagnosis. *Brucella spp.* were isolated from patients’ blood samples following confirmation of their consent. Collection of the animal samples was carried out by professional veterinary technologists in accordance with the ARRIVE guidelines^14^ and general local regulations and guidelines.

### Data source and statistical analysis

Epidemiological data on human brucellosis in Shaanxi Province, China, were obtained from the Disease Prevention and Control Information System of China and the Report Information Management System of Infectious Disease of China. According to related epidemiological indices, case numbers, incidence, time, region, age, and occupation distributions were sampled and downloaded. In parallel, the annual demographics of Shaanxi Province were extracted from the National Bureau of Statistics. The average incidence rates from 2008 to 2020 were defined as the total number of cases/total population × 100,000. The epidemiological data analysed were cleaned and processed with Excel 2016 software (Microsoft, Redmond, WA, USA). The case number, incidence rate, and constituent ratio (rate) were calculated to characterize the epidemiology of human brucellosis in Shaanxi Province. Furthermore, based on the diagnosis time of reported cases, the correlation between the onset time and the diagnosis interval of human brucellosis was explored.

Based on the brucellosis investigation guideline of “Diagnosis for Brucellosis (WS 269-2019)”^15^, epidemiological survey protocols (demographic characteristics (e.g., agender, age, nationality, live address), epidemiologic indexes (e.g., contact history, onset date, clinical manifestation, occupation, food exposure, and sick contacts), and serial serology assays (Rose-Bengal Plate Test (RBPT), serum tube agglutination test (SAT), and milk ring test (MRT)) were employed to investigate the outbreak of milk-borne brucellosis that occurred in Bin County in 2019 (Supplementary 1). A condensed epidemiological survey of goat farms was conducted, including farm size, breeding, introduction, and abortus situation. Data were analysed using SPSS 25 (Chicago, IL, USA) software, and *P* values < 0.05 were considered significantly different. The incidence rate and geographic regions of human brucellosis and the distribution profile of strains were displayed using ArcGIS 10.8 Software (ESRI Inc.; Redlands, CA, USA).

### Collecting samples for bacteriology testing

All 77 strains in this study were obtained from passive surveillance, all of them from investigation and disposal of human brucellosis epidemic events. There were 19 strains from 1958 to 2008, and their sampling amount was unknown; 16 strains were from human blood, and three were from deer (two from deer blood, and one from the deer abortus foetus). The remaining 58 strains were recovered from 157 blood samples from patients with brucellosis who presented with fever (or sweats, fatigue, and joint muscle pain) and SAT ≥ 1:100+ + during 2014–2020. The 157 samples were sampled from eight different cities (districts), including Yulin (n = 54), Yan’an (n = 37), Weinan (n = 27), Xianyang (n = 18), Baoji (n = 6), Tongchuan (n = 12), Shangluo (n = 1), and Hancheng (n = 2). Moreover, 145 farmers, eight students, two veterinarians, one chef, and one driver were included.

### Isolation, and identification of strains

Isolation and culture of clinical strains were performed according to standard procedures in a biological safety II (p2) laboratory^16^. Briefly, five (~ 10 mL) blood samples were collected from each suspected patient and injected into a biphasic culture bottle (Germany, bioMérieux; China, Barrett Biotechnology (Zhengzhou) Co., Ltd.) in biosafety combination and incubated at 37 °C with 5% CO_2_ for 2–4 weeks. Suspected *Brucella* spp. clones were then further purified. All isolates were identified by conventional biotyping procedures. *B. melitensis* 16 M (BM), *B. abortus* 544 (BA), and *B. suis* 1330 (BS) were used as standard control strains. Identification reagents and standard products were purchased from the Veterinary Drug Supervision Institute of China. A total of 77 *Brucella* strains were obtained from patient and deer blood samples from nine cities (districts) in Shaanxi Province. *Brucella* strains were incubated for 84 h with plate medium and subsequently heat inactivated at 80 °C for 20 min. DNA preparation from the different strains was performed following the recommendations provided in a bacterial genome kit (Qiagen, Hilden, Germany). The extracted DNA was stored at − 20 °C until use.

### MLST genotyping of strains

MLST genotyping was performed according to a method described previously^12^. Briefly, polymerase chain reaction (PCR) amplification was performed in a 40 ml amplification system; 5 ml of purified PCR products was sequenced and assembled, and sequence data of each locus were aligned using MEGA 6.0 software according to published MLST sequences in GenBank (accession numbers AM694191-AM695630)^17^. *B. melitensis* 16 M, *B. abortus* 544, and *B. suis* 1330 reference strains were used as positive controls. A minimum spanning tree (MST) was constructed by BioNumerics software (unweighted pair group method using arithmetic averages) (version 7.5; Applied Maths, Belgium) based on 477 *Brucella* strains (77 reported here and 400 from 17 other provinces (including all Chinese strains in PubMLST through December 2020)) (Table S1) collected from the main brucellosis epidemic regions in China.

## Results

### Human brucellosis epidemic profile during the 1951–2020 period

**Figure 1:**
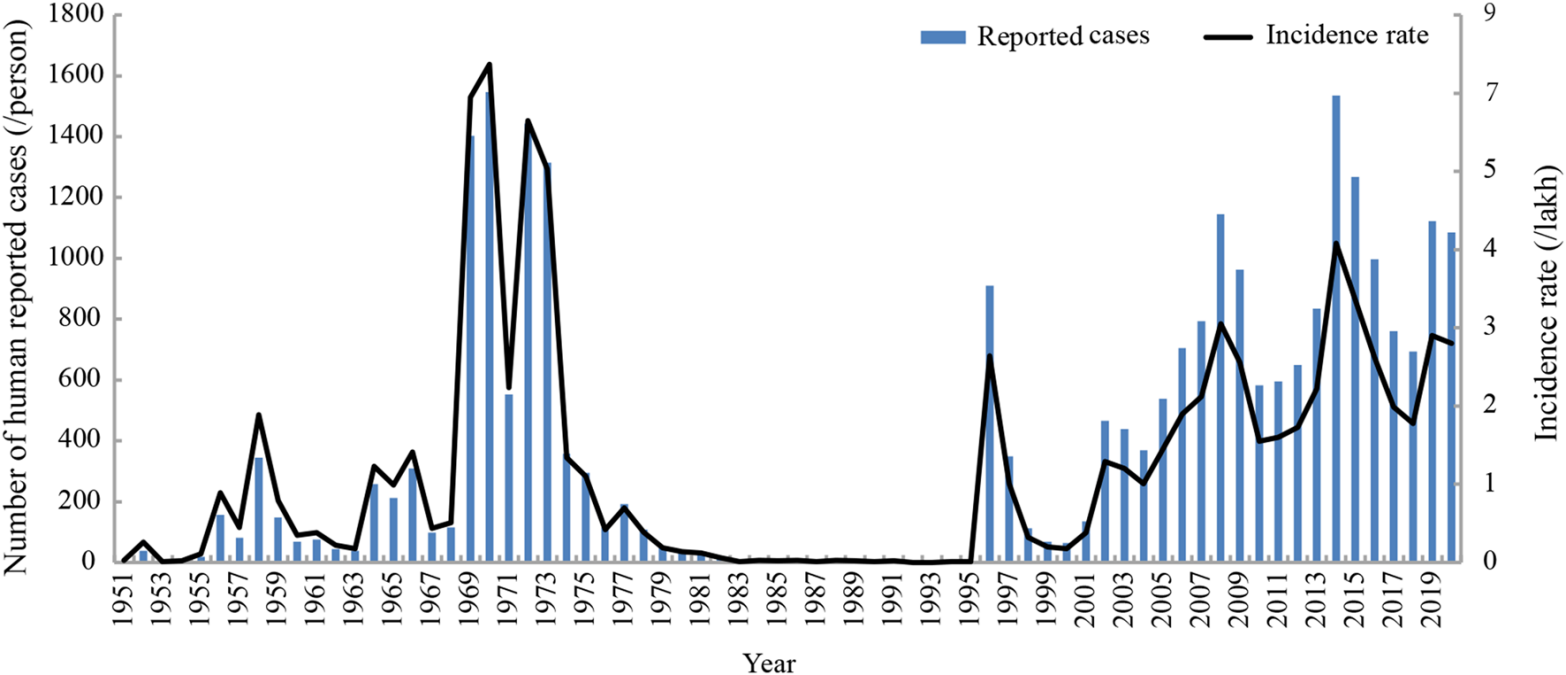
Annual reported cases and incidence rate (/100,000 people) of human brucellosis in Shaanxi Province during 1951–2020.

The human brucellosis epidemic in Shaanxi Province was divided into three stages: an epidemic period during 1951–1982, a control period during 1983–1995, and a re-emergence period during 1996–2020 (Fig. 1). The first human brucellosis case in Shaanxi Province was reported in 1951. Subsequently, cases increased year by year, and the number of reported cases reached a peak in 1970 and began to decline in 1974. From then until 1983, human brucellosis was basically controlled before re-emerging in 1996, and case numbers have continuously increased during the past two decades. A systematic survey of human brucellosis in Shaanxi Province was performed in 2008. A total of 12,215 human brucellosis cases were reported during 2008–2020, corresponding to an annual incidence rate range of 1.55/100,000–4.08/100,000, with an annual average incidence rate of 2.48/100,000. The annual case numbers were 2008, 1145 cases; 2009: 963 cases; 2010: 583 cases; 2011: 596 cases; 2012: 649 cases; 2013: 835 cases; 2014: 1535 cases; 2015: 1267 cases; 2016: 994; 2017: 759 cases; 2018: 681 cases; 2019: 1122; and 2020: 1086 cases (Table 1). From 2002 to 2020, the incidence rate of human brucellosis in this region steadily increased (Fig. 1).

**Table 1:** Epidemiological characteristics of human brucellosis cases in Shaanxi, 2008–2020.

Variables	Years	Number of cases (incidence rate (/lakh)	Gender distribution (case numbers)
			Male/female (incidence rate (/lakh))
Reported cases and gender profile	2008	1145 (3.05)	910/235 (4.70/1.30)
	2009	963 (2.56)	769/194 (3.96/1.07)
	2010	583 (1.55)	462/121(2.37/0.66)
	2011	596 (1.60)	467/129 (2.42/0.72)
	2012	649 (1.73)	513/136 (2.66/0.75)
	2013	835 (2.22)	636/199 (3.28/1.10)
	2014	1535 (4.08)	1220/315 (6.28/1.73)
	2015	1267 (3.36)	955/312 (4.89/1.71)
	2016	994 (2.62)	799/195 (4.08/1.06)
	2017	759 (1.99)	579/180 (2.94/0.98)
	2018	681 (1.78)	509/172 (2.59/0.93)
	2019	1122 (2.90)	832/290 (4.17/1.55)
	2020	1086 (2.80)	791/295 (3.95/1.57)
	Total	12,215 (2.48)	9442/2773 (3.72/1.17)

	Occupation	Reported cases	Constitution ration (%)
Occupation distribution profile	Farmers	10,815	88.54
	Students	261	2.14
	Staff	143	1.17
	Herdsman	103	0.84
	Scattered children	134	1.1
	Housekeeping, housework, unemployment	111	0.91
	Veterinary	93	0.76
	Workers	78	0.64
	Retired people	71	0.58
	Business personnel	50	0.41
	Civil servant and staff	39	0.32
	Livestock slaughter and livestock product processing personnel	40	0.32
	Others	277	2.27
	Total	12,215	100

	Ages group	Cases	Constitution ration (%)
Age distribution profile	19 days~	245	2.01
	10~	280	2.29
	20~	972	7.96
	30~	1519	12.44
	40~	3084	25.25
	50~	3694	30.24
	60~	1972	16.14
	70~	411	3.36
	≥ 80~	38	0.31
	Total	12,215	100

### Seasonal and occupational distribution of human brucellosis

Human brucellosis cases were reported every month of the year, and the period with the highest incidence was from March to August, accounting for 69.25% of the total number of cases; the peak incidence was from April to July, accounting for 51.26% of the total number of cases (Table S2).

The Cochran-Armitage test showed that the incidence rate exhibited a trend of first increasing rather than decreasing (χ^2^ trend 252.497, *P* < 0.001), and the highest incidence rate was in June. Moreover, 88.54% of the total number of cases were farmers (10,815/12,215), followed by students (261 cases), accounting for 2.14% of the total number of cases (Table 1).

### Age and gender distribution of human brucellosis

The 12,215 cases of brucellosis were distributed among various age groups, with a median age of 49 years. The maximum age of onset was 90 years, and the youngest was 19 days. The 50–59-year-old age group had more cases, accounting for 30.24% of the total, followed by 40–49-year-olds with 25.25% of total cases and the 30–39-year-old and 60–69-year-old age groups together accounting for 28.58% of the cases (Table 1). The Cochran-Armitage test showed that with age, the incidence rate exhibited a trend of first increasing than declining (χ^2^ trend 23,774.560, *P* < 0.001), and the highest incidence rate was in the 50–59 group. Brucellosis predominantly occurred in males, accounting for 77.30% of the total cases, and the ratio of males to females was 3.40:1. The incidence of brucellosis between men and women was statistically significant (χ^2^ = 3220.715, *P* < 0.001) (Table 1).

### Regional distribution profile of human brucellosis

**Figure 2:**
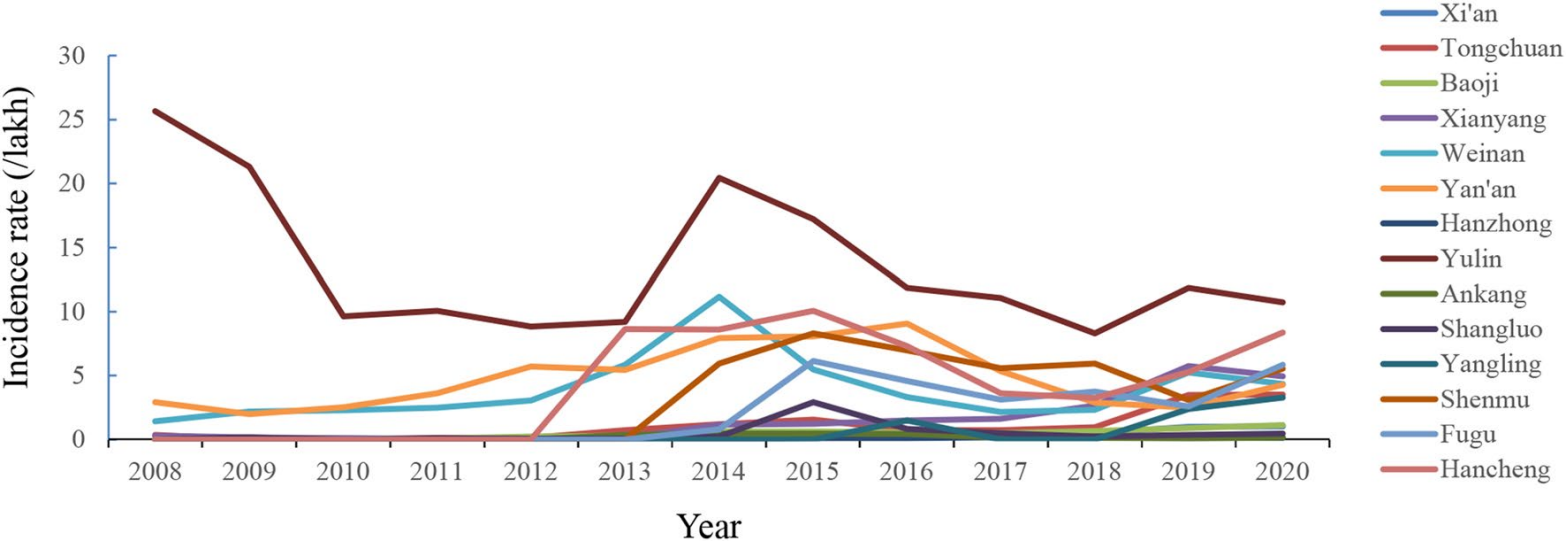
Regional incidence rate trend of human brucellosis in Shaanxi Province during 2008–2020.

**Figure 3:**
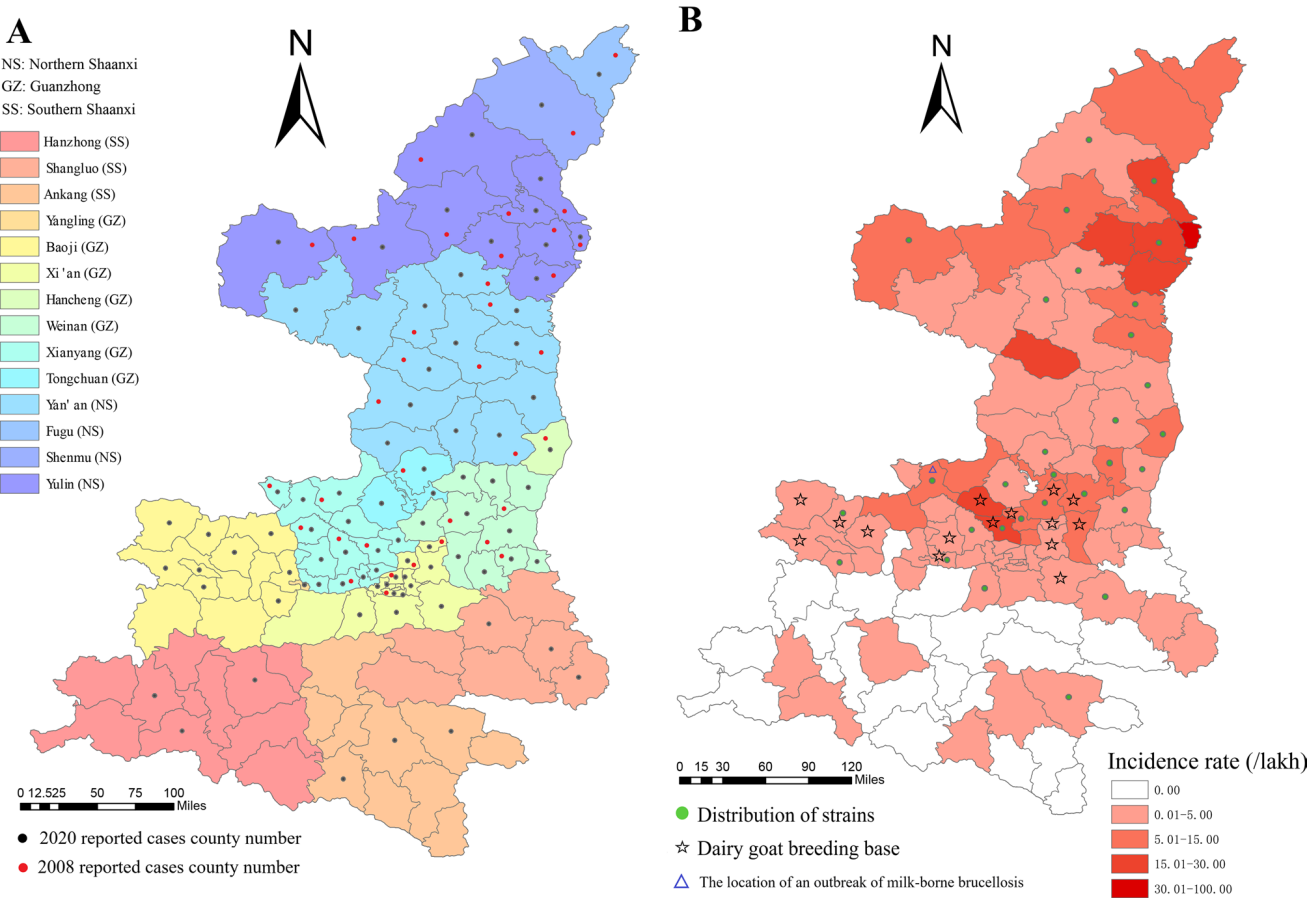
(**A**) Reported regional distribution characteristics of two different years (2008 and 2020) of human brucellosis cases in Shaanxi Province. (**B**) Regional incidence rate of human brucellosis and 77 strain distribution profile of Shaanxi Province during 2008–2020. Map was generated using ArcGIS 10.8 (https://desktop.arcgis.com).

During 2008–2012, most human brucellosis cases were reported from Yulin (176.00/100,000), Yan'an (62.22/100,000), and Weinan (51.25/100,000); there were no cases reported in Fugu, Yangling, Shenmu, Hancheng, or Hanzhong, while only a few cases were reported in other regions of Shaanxi Province (Fig. 2). No cases were reported in Yangling City during the periods 2013–2015 and 2017–2018. Cases occurring in all other counties (cities/districts) between 2013 and 2020 were reported. The incidence rate in Yulin was 176.00/100,000, which was higher than that in any other area. The lowest incidence rates were observed in Hanzhong, Ankang, and Xi'an, at 0.97/100,000, 2.19/100,000, and 4.18/100,000, respectively (Fig. 2). The county numbers of reported cases increased from 36 in 2008 to 84 in 2020 (Fig. 3A). The human brucellosis epidemic had an apparent geographic expansion trend from northern Shaanxi to Guanzhong and southern Shaanxi in this Province (Fig. 3A). However, the incidence rate declined steadily in northern Shaanxi regions; in contrast, the incidence rate has been increasing yearly in some Guanzhong and southern Shaanxi regions, such as Hancheng city and Xianyang (Fig. 3B).

### The onset of illness and diagnosis interval

Based on the diagnosis time analysis, reported cases of human brucellosis were concentrated from March to August, reaching a peak in June, while the peak of brucellosis onset was concentrated from February to July, reaching a peak in May. From the perspective of temporal distribution, the peak diagnosis time of reported cases occurs one month later than the peak in the onset of brucellosis (Fig. S1).

### Diagnosis interval temporal profile

**Figure 4:**
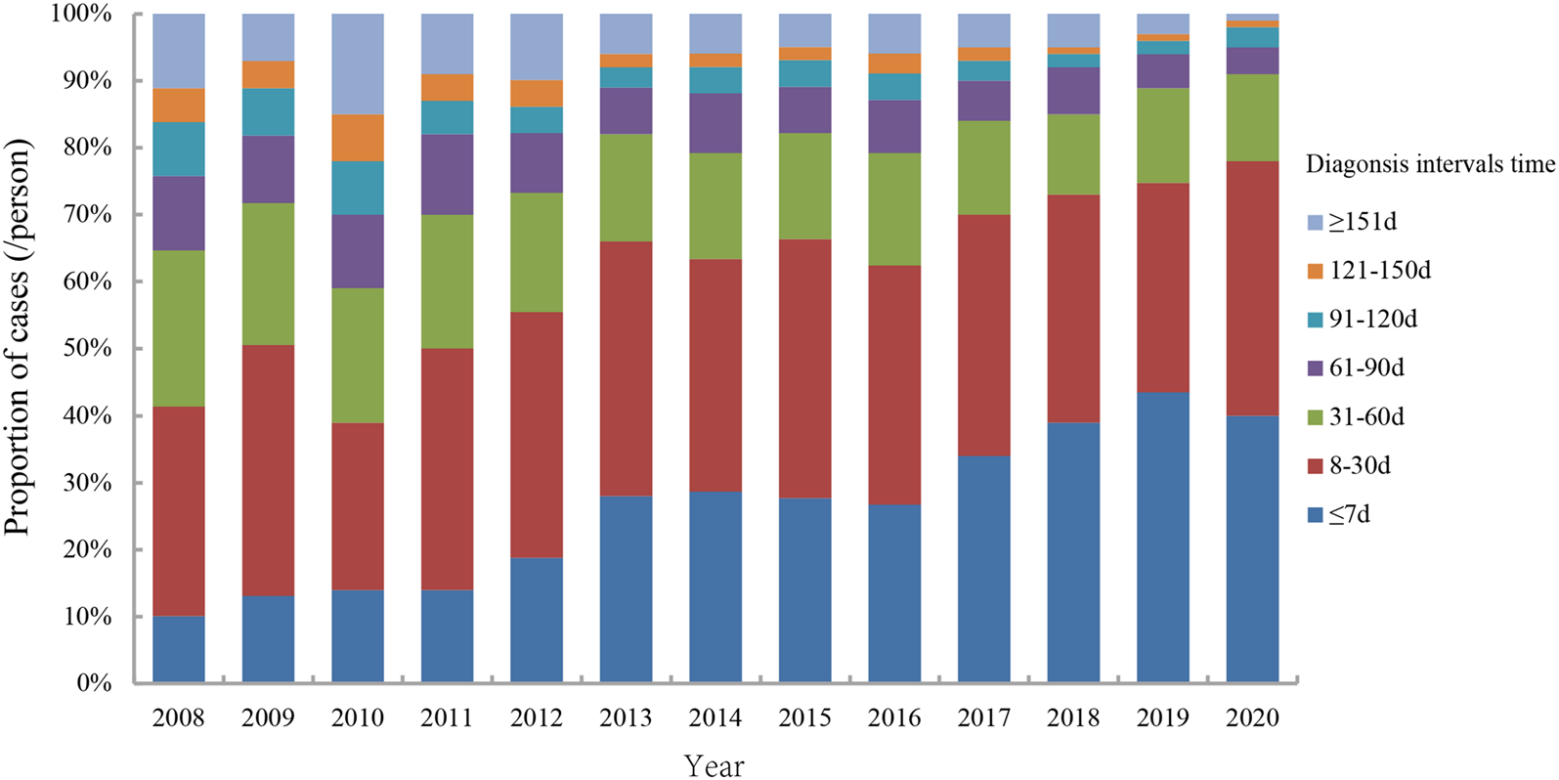
Diagnosis interval temporal profile of human brucellosis cases during 2008–2020.

Among the 12,215 cases of brucellosis reported, the time interval from onset to diagnosis ranged from 0 to 3693 days, and the median time interval was 22 days. The median interval between brucellosis incidence and diagnosis reported in Shaanxi Province from 2008 to 2020 showed an overall decline (Fig. 4).

### Conventional biotyping and distribution of the 77 *Brucella* isolates

All 77 isolated strains were identified as *Brucella* spp., and the 3 species identified were *B. melitensis* (n = 74) (11 in *B. melitensis* bv. 1, 7 in *B. melitensis* bv. 2, 46 in *B. melitensis* bv. 3, and 10 in *B. melitensis* variant), *B. abortus* bv. 3/6 (n = 2), and *B. suis* bv. 1 (n = 1). *B. melitensis* bv. 3 was the dominant species, accounting for 59.74% (46/77) (Table 2). These strains were isolated during 1958–2020. Of the 77 strains isolated, 74 *B. melitensis* were obtained from human blood, and 3 strains were isolated from deer. All 77 strains were distributed in nine different cities (including 30 counties (districts/cities)), 22 in Yulin, 17 in Weinan, 15 in Xianyang, 12 in Yan'an, 4 in Tongchuan, 3 in Xi'an, 2 in Baoji, 1 in Ankang, and 1 in Shangluo City (Fig. 3B).

**Table 2:** Conventional biotyping identification of 77 *Brucella* isolates from Shaanxi Province.

Strains	CO_2_ requirement	H_2_S production	Serum agglutination tests				Bacteriostatic tests (dye)		Phage lysis tests			Number	Interpretation
			Positive serum	A	M	R	Bufothionine	Fuchsin	BK_2_	10^4^ Tb	WB		
16 M	−	−	+	−	+	−	+	+	+	−	−	1	*B. melitensis* 1
63/9	−	−	+	+	−	−	+	+	+	−	−	1	*B. melitensis* 2
Ether	−	−	+	+	+	−	+	+	+	−	−	1	*B. melitensis* 3
544	±	+	+	+	−	−	−	+	+	+	+	1	*B. abortus* 1
1330	−	+	+	+	−	−	+	−	+	−	+	1	*B. suis* 1
Tested strains	−	−	+	−	+	−	+	+	+	−	−	11	*B. melitensis* 1
	−	−	+	+	−	−	+	+	+	−	−	7	*B. melitensis* 2
	−	−	+	+	+	−	+	+	+	−	−	46	*B. melitensis* 3
	−	−	+	+	+	+	+	+	+	−	−	10	*B. melitensis* variant
	−	−	+	+	−	−	+	+	+	+	+	2	*B. abortus* 3/6
	−	+	+	+	−	−	+	−	+	−	+	1	*B. suis* 1

+ , positive; −, negative; ± , some strains were positive.

### MLST genotyping

**Figure 5:**
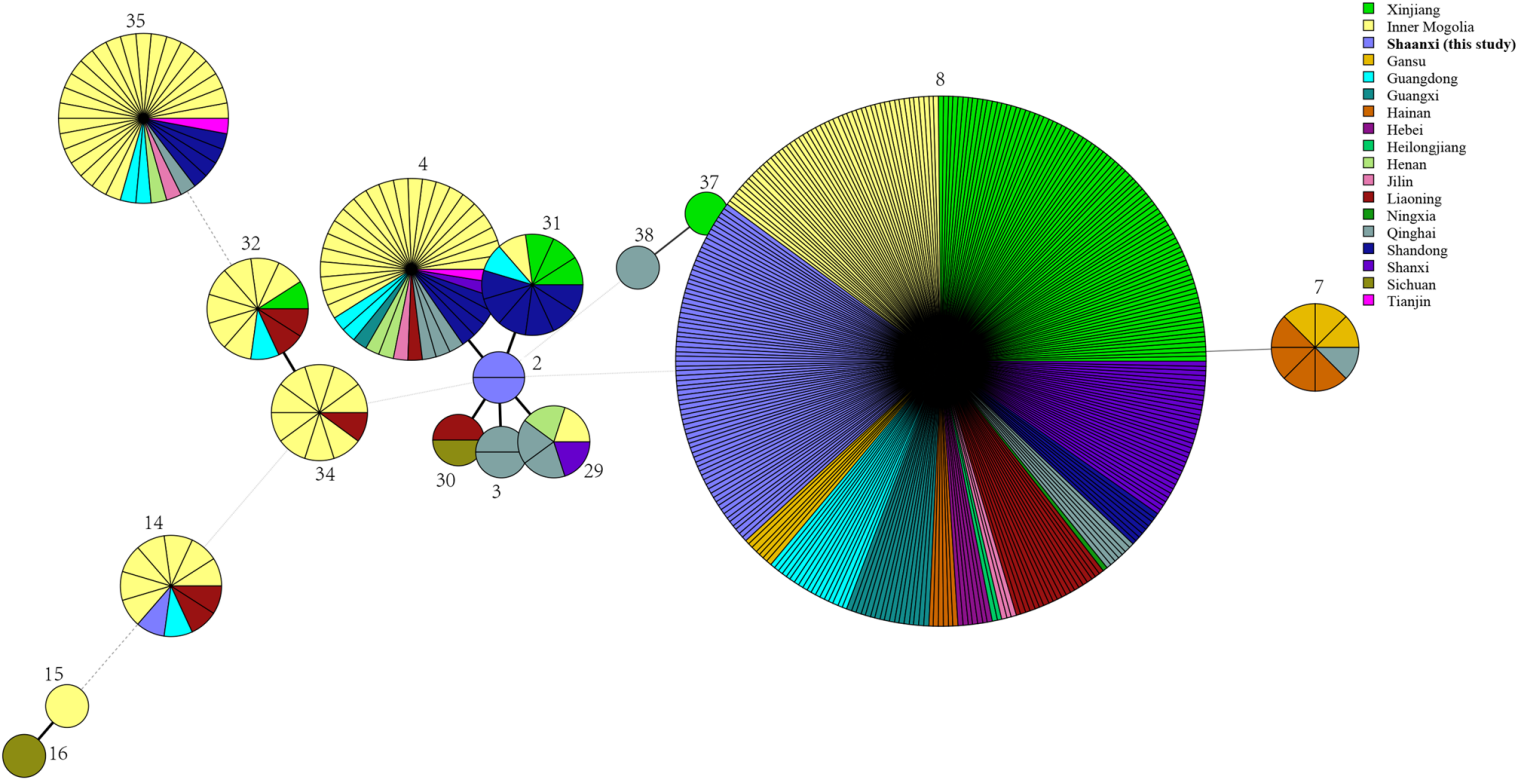
MST analysis based on MLST data in 477 *Brucella* *melitensis* strains (77 reported here and the remaining strains from China). Numbers in the figure refer to the ST type of strains analysed. Each ST is represented by a circle, the size of which reflects the number of isolates.

All isolates were further analysed by MLST, and three known STs were identified. *B. suis* belonged to ST14 (1-6-4-1-4-3-5-2–1; n = 1); *B. abortus* belonged to ST2 (2-1-2-2-1-3-1-1; n = 2), and all *B. melitensis* strains were identified as ST8 (3-2-3-2-1-5-3-8-2; n = 74). ST2 was found in 1958 (n = 1) and 2005 (n = 1); ST14 was observed in 1974 (n = 1). ST8 was observed during 1958–2020, including 1958 (n = 2), 1973 (n = 1), 1978 (n = 9), 1979 (n = 1), 2008 (n = 3), 2014 (n = 7), 2015 (n = 8), 2016 (n = 7), 2017 (n = 7), 2018 (n = 12), 2019 (n = 12), and 2020 (n = 5) (Table 3). In the present study, ST8 was predominately a clonal population and was observed in all nine regions and all examined periods (Table 3). MST analysis showed that ST8 from this study was shared with strains from 14 other provinces, including Inner Mongolia, Xinjiang, Shanxi, Shandong, Qinghai, Liaoning, Hebei, Tianjin, Jilin, and Gansu (Fig. 5). ST14 was distributed in Shaanxi, Inner Mongolia, Hainan, and Guangdong Provinces. However, ST2 formed a unique clone in this study (Fig. 5).

**Table 3:** ST distribution profiles of 77 *Brucella* strains in Shaanxi Province.

Key	Species/Biovar	ST	Numbers	Host	Year
1	*B. suis bv. 1*	14	1	Deer	1974
2	*B. abortus bv. 3/6*	2	2	Human	1958, 2005
3	*B. melitensis bv. 1*	8	11	Human, Deer	1973, 1978, 1979, 2017, 2018, 2020
4	*B. melitensis bv. 2*		7	Human	1958, 1978
5	*B. melitensis bv. 3*		46	Human	2008, 2014–2020
6	*B. melitensis variant*		10	Human	2016–2020

ST, sequence types.

## Discussion

At present, human brucellosis has been reported in all 32 mainland provinces of China^18^. Our study showed that the human brucellosis epidemic in Shaanxi Province was divided into three stages: an epidemic period during 1951–1982 (peaking during 1969–1975), a controlled period 1983–1995, and a re-emergence period 1996–2020. Our conclusion is consistent with previous reports that human brucellosis incidence in China was divided into three stages: high incidence (1950s–1960s), decline (1970s–1980s), and re-emergence (1990s–2000s)^19^. During 1950–2000, more than 80% of total cases were reported in husbandry-developed northern regions, such as Inner Mongolia, Xinjiang, and Shanxi Provinces^19^. Shaanxi Province is in the centre of northern China and includes Inner Mongolia, Gansu, Ningxia, and Shanxi; these nearby provinces have all reported a high prevalence of brucellosis, and the epidemic in human brucellosis peaked later than in the northern provinces. Although the annual average incidence rate (2.48/100,000) of human brucellosis in this region is significantly lower than that in other northern regions^20^, the epidemiological features have obviously changed. The county numbers of reported cases increased from 36 in 2008 to 84 in 2020. Human brucellosis had an apparent north-to-south geographic expansion trend. Similarly, previous reports concerning the spatiotemporal expansion of brucellosis in Shaanxi Province showed that human cases slowly extended towards the southern region, with significant seasonal fluctuations^10^. Moreover, the incidence rate has declined in previously epidemic northern Shaanxi regions (Yulin, Yan'an, and Weinan Cities), in contrast to the yearly increases in some emerging Guanzhong and southern Shaanxi epidemic regions, such as Hancheng and Xianyang. Similarly, since 2015, the incidence of human brucellosis has shown opposite trends in northern and southern China; rates in northern China have fallen, while rates in southern China have increased^7^. The increasing incidence rate in Guanzhong and southern Shaanxi regions may be related to the development of large goat (sheep) farming operations. Shaanxi is the country’s largest dairy goat production base and is known as the “Hometown of Dairy Goats in China.” After 2018, preliminary estimates indicated that approximately 325 dairy goat industry construction projects had been implemented in 3 demonstration counties for the entire industrial chain of dairy goats and 12 base counties^21^. Moreover, it is estimated that by the end of 2018, there will be two million dairy goats in the province, a yearly increase of 17.6%^21^. With economic and social development, large-scale animal husbandry has emerged, leading to the rapid spread of human brucellosis in China. Surveillance and prevention of this disease are significant challenges^22^. We suggest that some control and preventive strategies should be implemented; these include but are not limited to strict control of infected animal (sheep and goats) trade and migration (transfer), implementation of a strict introduction and quarantine system, mass vaccination of livestock in high incidence rate regions of human brucellosis, slaughter or elimination of infected animals, and regular serology surveillance of domestic animals.

Seasonality features included an incidence peak in March to August. Moreover, the 50–59-year-old age group had more cases; the ratio of males to females of 3.40:1 was similar to the results of previous studies^16,23^. Another study showed that a total of 1149 human brucellosis data points were collected during 2014–2018 in Huludao (China), with a mean age of 49.59 ± 13.14 years, and 75.7% were male^24^.

It is noteworthy that 261 student cases were recorded, ranking second among all occupations. Moreover, 68 student cases of brucellosis were reported in rural regions from 2019 to 2020. Field epidemiological surveys showed that consumption of raw milk was the main reason for these brucellosis cases in the student population. Similarly, a previous research study showed that contact with infected animals and consumption of raw milk and milk products were the main risk factors associated with brucellosis in Saudi Arabia^25^. In Germany, unpasteurized milk products were most frequently identified as sources of brucellosis infection during 2006–2018^26^. Similarly, outbreaks of brucellosis related to the consumption of unpasteurized camel milk were reported in a rural area in Qatar^27^. A study in an Iranian children’s referral hospital showed that a history of ingestion of raw or unpasteurized dairy products was present in 88% of cases (N = 38), and 11 patients (26%) had contact with a suspected animal carrier^28^. A global systematic review and meta-analysis of contamination of milk and dairy products by *Brucella* species showed that the highest prevalence of *Brucella* contamination in dairy products was noted in buffaloes (25.91%) and goats (17.90%); moreover, decreasing poverty and an increase in the level of education in societies could reduce the prevalence of *Brucella* spp. in dairy products^29^. We suggest that proper training and education be introduced in the student population to increase awareness of the dangers of consuming unpasteurized dairy products. In addition, parents are urged to establish correct nutritional concepts and be informed that there is no significant difference in the nutritional value of pasteurized milk versus raw milk.

Our analysis showed that the peak diagnosis time of reported brucellosis cases is one month later than the peak in the onset of the disease. Moreover, the median interval between brucellosis incidence and diagnosis declined from 2008 to 2020. A related study from nearby Sichuan Province showed that the misdiagnosis rate of human brucellosis was high in newly affected counties, and the diagnosis was clearly delayed^30^. Because brucellosis is a re-emerging disease in some Guanzhong and southern Shaanxi regions, the suspicion index for human brucellosis in doctors was extremely low. Human brucellosis has similar clinical symptoms to many diseases, and this may lead to delay of diagnosis and increases in complications^31,32^. We suggest that physicians in brucellosis re-emerging areas should have more training and health education and that excluding brucellosis should be a priority once patients display brucellosis-like symptoms. Furthermore, strengthening the capacity of brucellosis laboratory diagnosis in hospitals and CDCs at the county level is necessary^30^.

*B. melitensis* strains were the dominant species in the examined periods, and *B. melitensis* biovar 3 was the predominant biovar, suggesting that infected sheep (goats) were the main source of infection for most human brucellosis cases in this province. Strains were mainly distributed in the northern Shaanxi and Guanzhong regions; fewer strains were observed in the southern Shaanxi regions. Further bacteriology surveillance in southern regions of Shaanxi Province is recommended. Our previous report confirmed that the geographic distribution of brucellosis has also evolved, with the movement of *B. melitensis* strains to the Guanzhong and southern Shaanxi regions^33^. Other research suggests that *B. melitensis* biovar 3 was the dominant species and was shown to be widespread in all countries along the Silk Road. Prevention and surveillance of the *B. melitensis* population is challenging^34–36^. Therefore, strengthening surveillance and control in infected sheep (goats) is warranted in this province. Subsequently, the genetic relatedness and population structure of *B. melitensis* were investigated using MLST. Although three known STs were identified, ST8 was the dominant clone and was observed in all nine regions and during the entire period examined. Strains from the ST8 clone population are widespread in China in Inner Mongolia, Xinjiang, Gansu, Qinghai, and Guangxi Provinces^17,19,37^. Another study showed that more isolates were clustered around ST8 during the re-emergence stage (1990s–2010s)^38^. These data suggest potential epidemiological links among these regions. Furthermore, a study of 66 isolates collected from sheep and yaks from Northwest China (Inner Mongolia, Xinjiang, Qinghai, and Gansu Provinces) during 2015 and 2016 showed that ST8 was the dominant genotype in those *B. melitensis* isolates, and this *Brucella* genotype is widespread in Northwest China^39^. We suggest that further MLVA (multiple-locus variable-number tandem repeat analysis)^40^ and WGS-SNP (whole-genome sequencing-single-nucleotide polymorphism)^41,42^ should be applied to investigate the source of infection of human brucellosis in Shaanxi Province. The purpose of such an investigation should target measures of control and prevention of human brucellosis. Moreover, strengthened surveillance of animal brucellosis and banning infected sheep (goat) transfer are optimal control strategies.

Moreover, our study has some limitations. First, the data used were extracted from a passive public health surveillance system that might be influenced by multiple pertinent factors, such as case definitions and laboratory facilities, ability of inspection technicians, and physician awareness of the disease. Second, the geographic distribution of the isolated strains was imbalanced, and strains obtained from animals were uninvolved in our study, which may partly explain the epidemiological features of strains. Third, surveys of the source of infection of human brucellosis are lacking because MLST has low discriminatory power for closely related strains. Therefore, further molecular epidemiology characterized using MLVA and WGS-SNPs to strains is necessary.

## Conclusion

In summary, based on notifiable surveillance data in Shaanxi Province, China, during 2008–2014, we found that the epidemiological characteristics of human brucellosis in this region were obviously altered. The affected areas of this disease have expanded from northern Shaanxi to Guanzhong and southern Shaanxi in this Province, especially since 2008. The development of animal husbandry farming in the Guanzhong and southern Shaanxi regions is the main reason for the yearly human brucellosis increases. The only constant factor was that *B. melitensi*s biovar 3 belonging to the ST8 lineage was the overwhelming dominant population in this region during the entire examined period, indicating that sheep (goats) were the principal reservoirs for human brucellosis. We suggest that strict brucellosis control strategies in animals and humans should be implemented to meet requirements for disease control and prevention.

## Supplementary Information

Supplementary Information 1.

Supplementary Table S1.

Supplementary Table S2.

Supplementary Figure S1.

## Data Availability

All data generated or analysed during this study are included in this published article, and the supplementary information files will be freely available to any scientist for non-commercial purposes upon request to the corresponding author via email.

## Abbreviations

RBPT: Rose Bengal Plate Test
SAT: Serum tube agglutination test
MRT: Milk ring test
PCR: Polymerase chain reaction
MST: Minimum spanning tree
MLST: Multi-locus sequence typing
MLVA: Multiple-locus variable-number tandem repeat analysis
WGS-SNP: Whole-genome sequencing-single-nucleotide polymorphism
