# Supplementary material for: Changes in the epidemiological characteristics of human brucellosis in Shaanxi Province from 2008 to 2020
Source: Sci Rep. 2021 Aug 30;11:17367. doi: 10.1038/s41598-021-96774-x (PMC8405659; doi:10.1038/s41598-021-96774-x)
Supplement: Supplementary file 1 — Supplementary Information 1. [file 41598_2021_96774_MOESM1_ESM.docx]

**Supplementary 1 Investigate the outbreak of milk-borne brucellosis in Bin County in 2019**

**Epidemiology survey** **on the outbreak of milk-borne brucellosis that occurred in Bin County**

On October 18, 2019, one human brucellosis case (three-year-old, SAT>100++) was reported in Bin County in the Notifiable Infectious Diseases Reporting Information System; the patient had a fever (temperature unknown) and night sweat clinical manifestations. Moreover, the patient had a history of ingested goat milk bought from one goat farm. A total of 419 blood samples from pertinent individuals (villagers and workers of goat farms) with this case were collected and detected by the Rose-Bengal Plate Test (RBPT) and serum tube agglutination test (SAT). A comprehensive epidemiological survey of 419 individuals was performed, and investigation items included demographic characteristics (e.g., gender, age, nationality, live address) and epidemiologic data (e.g., contact history, clinical manifestation, occupation, food exposure, and sick contacts). A total of 499 (milks and serum) samples from the farm goat were collected and tested by the MRT, RBPT, and SAT. A condensed epidemiological survey of goat farms was conducted, including farm size, breeding, introduction, and abortus situation.

**The outbreak of milk-borne brucellosis analysis**

Based on epidemiological and laboratory data, 43 out of 419 samples were seropositive against brucellosis in Bin County. The 42 cases out of 43 had a history of ingesting goat milk from the above goat farm, and one case (worker) was found on this farm. Twenty-five cases occurred in patients under 12 years old, accounting for 28.0% (12/43). All 43 cases were distributed in seven townships and 34 villages. Moreover, 59 goats (11.8%, 59/499) were diagnosed with brucellosis. The survey showed that a ram was introduced to the farm on May 30, 2019. On July 28, 2019, serology screen testing showed that 44 goats (a ram and 43 ewes) were seropositive against brucellosis. These data indicate that the introduced infected ram was the main risk for serial brucellosis outbreak events, but further investigation into the source of infection is needed.
