# Supplementary material for: Changes in the epidemiological characteristics of human brucellosis in Shaanxi Province from 2008 to 2020
Source: Sci Rep. 2021 Aug 30;11:17367. doi: 10.1038/s41598-021-96774-x (PMC8405659; doi:10.1038/s41598-021-96774-x)
Supplement: Supplementary file 3 — Supplementary Table S2. [file 41598_2021_96774_MOESM3_ESM.docx]

Table S2 Seasonal distribution of brucellosis in Shaanxi province, 2008 – 2020.

| Month | Cases | Constitution ration (%) |
| --- | --- | --- |
| 1 | 660 | 5.4 |
| 2 | 741 | 6.1 |
| 3 | 1046 | 8.6 |
| 4 | 1453 | 11.9 |
| 5 | 1619 | 13.3 |
| 6 | 1676 | 13.7 |
| 7 | 1514 | 12.4 |
| 8 | 1151 | 9.4 |
| 9 | 727 | 5.9 |
| 10 | 662 | 5.4 |
| 11 | 469 | 3.8 |
| 12 | 497 | 4.1 |
| Total | 12215 | 100.0 |
