# Supplementary figures and images for: Changes in the epidemiological characteristics of human brucellosis in Shaanxi Province from 2008 to 2020
Source: Sci Rep. 2021 Aug 30;11:17367. doi: 10.1038/s41598-021-96774-x (PMC8405659; doi:10.1038/s41598-021-96774-x)

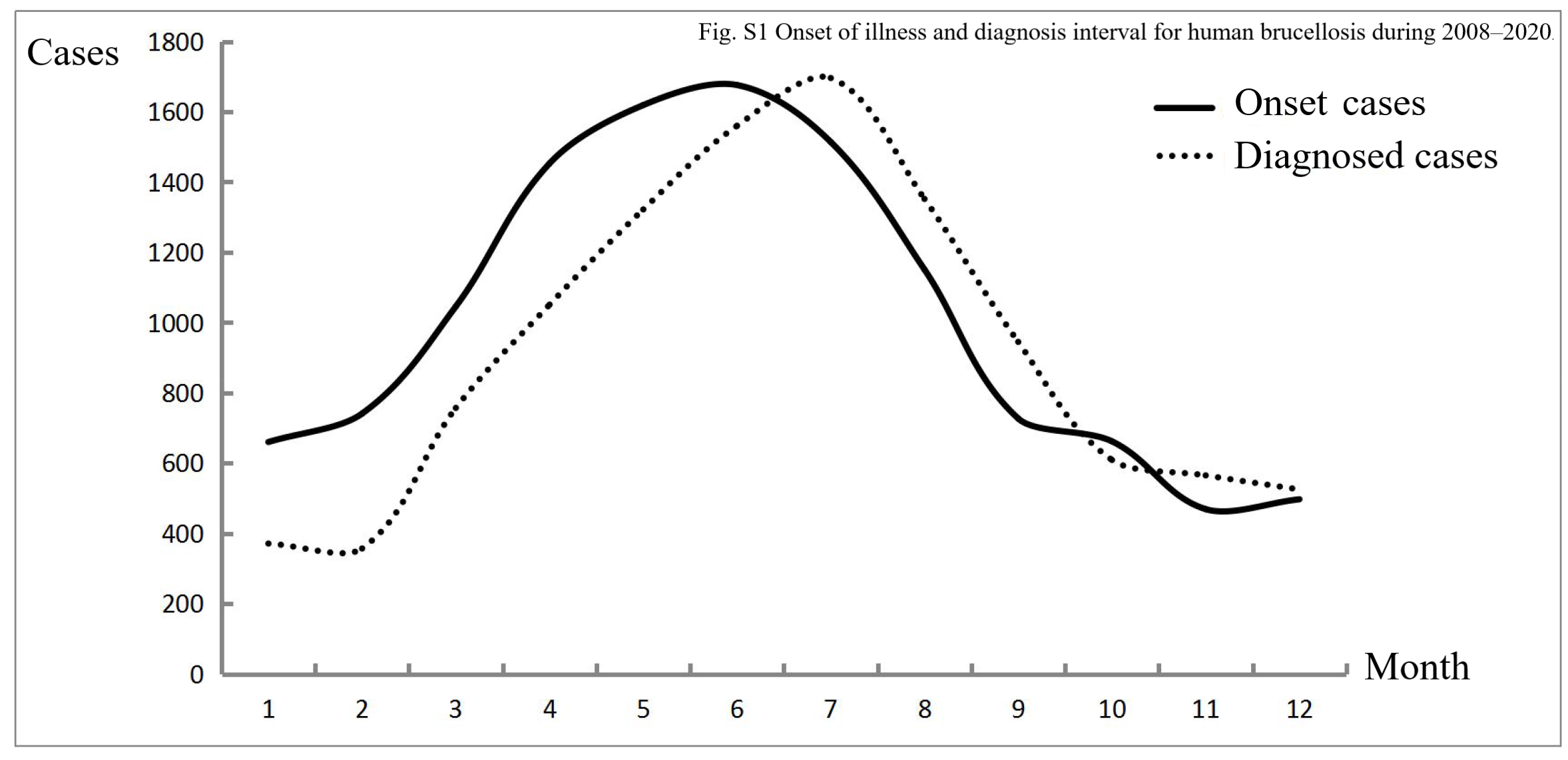

Supplement: Supplementary file 4 — Supplementary Figure S1. [file 41598_2021_96774_MOESM4_ESM.tif]
